# EHMTI-0249. Headache attributed to hypertension in chronic migraine patients

**DOI:** 10.1186/1129-2377-15-S1-C18

**Published:** 2014-09-18

**Authors:** O Grosu, I Moldovanu, S Odobescu, L Rotaru

**Affiliations:** 1 Neurology, Institute of Neurology and Neurosurgery, Chisinau, Moldova

## Background

Hypertension is frequent in chronic migraine patients and could be a factor that promotes chronification (Bigal, 2008). In these patients could be a secondary headache named “headache attributed to hypertension”.

## Aim

to detect the presence of “headache attributed to hypertension” in chronic migraine patients with high blood pressure (HBP).

## Method

Study sample include 60 chronic migraine patients with associated arterial hypertension (MwHBP) and 30 patients with hypertension without migraine (HBPw/oM). All the patients underwent neurological examination, ambulatory blood pressure monitoring. Headache was established according to IHS criteria (2004,2013).

## Results

In the MwHBP group 65% of patients presented a second type of headache corresponding to “headache attributed to hypertension” criteria compared to 33% in the HBPw/oM group. This type of headache in MwHBP group was unilateral (60% vs. 23.1%, p<0.05), pulsating (60% vs. 17.9%, p<0.05), in any part of the day (80% vs. 23.1%, p<0.05), with the combination of two or more associated symptoms (100% vs. 0%) compared with HBPw/oM group - predominantly on the morning, with isolated associated symptoms (30% - just photophobia vs. 0%, 40% - just phonophobia vs. 0%, 30% - just nausea vs. 0%, p<0.05).

## Conclusion

In the chronic migraine patients with associated hypertension could be detected two types of headaches. The first one is migraine and the second - “headache attributed to hypertension”, which is more frequent than in the hypertension without migraine group (65% vs. 33%) and preserved the migrainous features.

No conflict of interest.

